# Sampling solution traces for the problem of sorting permutations by signed reversals

**DOI:** 10.1186/1748-7188-7-18

**Published:** 2012-06-15

**Authors:** Christian Baudet, Zanoni Dias, Marie-France Sagot

**Affiliations:** 1 Laboratoire Biométrie et Biologie Evolutive, Université de Lyon, Université Lyon 1, CNRS, Villeurbanne, UMR5558, France; 2 INRIA Grenoble-Rhône-Alpes, team BAMBOO, 655 avenue de l’Europe, 38334 Montbonnot Cedex, France; 3 Institute of Computing, University of Campinas, Campinas - SP, Brazil

**Keywords:** Reversals, Traces, Sampling, Genome rearrangement

## Abstract

**Background:**

Traditional algorithms to solve the problem of sorting by signed reversals output just one optimal solution while the space of all optimal solutions can be huge. A so-called trace represents a group of solutions which share the same set of reversals that must be applied to sort the original permutation following a partial ordering. By using traces, we therefore can represent the set of optimal solutions in a more compact way. Algorithms for enumerating the complete set of traces of solutions were developed. However, due to their exponential complexity, their practical use is limited to small permutations. A partial enumeration of traces is a sampling of the complete set of traces and can be an alternative for the study of distinct evolutionary scenarios of big permutations. Ideally, the sampling should be done uniformly from the space of all optimal solutions. This is however conjectured to be *♯*P-complete.

**Results:**

We propose and evaluate three algorithms for producing a sampling of the complete set of traces that instead can be shown in practice to preserve some of the characteristics of the space of all solutions. The first algorithm (RA) performs the construction of traces through a random selection of reversals on the list of optimal 1-sequences. The second algorithm (DFALT) consists in a slight modification of an algorithm that performs the complete enumeration of traces. Finally, the third algorithm (SWA) is based on a sliding window strategy to improve the enumeration of traces. All proposed algorithms were able to enumerate traces for permutations with up to 200 elements.

**Conclusions:**

We analysed the distribution of the enumerated traces with respect to their height and average reversal length. Various works indicate that the reversal length can be an important aspect in genome rearrangements. The algorithms RA and SWA show a tendency to lose traces with high average reversal length. Such traces are however rare, and qualitatively our results show that, for testable-sized permutations, the algorithms DFALT and SWA produce distributions which approximate the reversal length distributions observed with a complete enumeration of the set of traces.

## Background

### Permutations and reversals

When studying genome rearrangements, we can identify homologous markers with the integers 1,…,*n*, with a plus or minus sign to indicate on which strand they lie. By using this notation, we can represent by a *signed permutation* the order and the orientation of the genomic markers of one species in relation to those of another.

A subset of numbers ρ⊆{1,…,n} is said to be an *interval* of a permutation *π *if there exist *i*,*j*∈{1,…,*n*}, 0 <* i *≤* j *≤* n*, such that *ρ *= {|*π*_*i*_|,…,|*π*_*j*_|}, where *π*_*x*_ is the element which is in position *x* of the permutation *π*. If we write the elements of different intervals in increasing order (for example, {2,3,6,8}), we can compare them using lexicographic order.

Two intervals are said to *overlap* if they intersect but none is contained in the other. For example, if *π *= (1,−4,3,2,−5,−6), then *ρ*_1_ = {1,3,4} and *ρ*_2_ = {2,3,4} overlap, while *ρ*_3_ = {2,3,4,5} and *ρ*_4_ = {2,3} do not.

Given a permutation *π *and an interval *ρ* of *π*, we can apply a reversal on *π*, that is, an operation which reverses the order and flips the signs of the elements of *ρ*. If *ρ*={|*π*_*i*_|,…,|*π*_*j*_|}, then *π*∘*ρ*=(*π*_1_,…,*π*_*i*−1_,−*π*_*j*_,…,−*π*_*i*_,*π*_*j* + 1_,…,*π*_*n*_). Due to this, we can use *ρ *to denote a reversal.

A sequence of *k* + 1 permutations can be represented by a sequence *π*^0^,*π*^1^,…,*π*^*k *^of permutations. In such a sequence, every pair of consecutive permutations *π*^*i*^ and *π*^(*i* + 1)^(0 ≤* i *<* k*) are just one reversal apart (*i.e.*, we need just one reversal to transform *π*^*i*^ into *π*^(*i* + 1)^ or vice-versa).

### Sorting permutations by signed reversals

A sequence of reversals *ρ*_1_…*ρ*_*d*_ sorts a permutation *π*^0^ if *ρ*_*i*_ is an interval of $\pi^{0}\circ \rho_{1}\cdots \rho_{i-1}$ for all *i*, and $\pi^{0}\circ \rho_{1}\cdots \rho_{d}$ is the target permutation *π*^*d*^ (usually, *π*^*d*^ is the identity permutation *ι*_*n *_= (1,2,…,*n*)). A shortest sequence of reversals sorting a permutation is called an *optimal* sorting sequence. The length of such a sequence of reversals is called *reversal distance*, denoted by *d*(*π*^0^,*π*^*d*^) (or simply *d*(*π*^0^), when $\pi^{d}=\iota_{n}$). For example, if *π*^0^=(−3,2,1,−4) and $\pi^{d}=\iota_{4}$, one optimal sorting sequence is {1}{4}{2}{1,2,3} and *d*(*π*^0^,*π*^*d*^)=*d*(*π*^0^)=4.

The problem of finding an optimal sorting sequence under this model (henceforward denoted by HP) due to Hannenhalli and Pevzner [1] is called *Sorting Permutations by Signed Reversals* (SPSR) and has been the topic of a vast literature. The first polynomial algorithm with complexity *O*(*n*^4^) was proposed in 1999 by Hannenhali and Pevzner [2]. In 2001, Bergeron presented a quadratic algorithm [3]. In 2004, Tannier, Bergeron and Sagot developed the first sub-quadratic algorithm with complexity $O(n^{3/2}\sqrt{\log n})$[4], while a linear algorithm, by Bader, Moret, and Yan, can calculate the reversal distance in linear time [5].

More recently, Swenson *et al.*[6] proposed an O(nlogn+kn) algorithm for finding one optimal scenario, where *k* is the number of successive corrections which must be applied when the algorithm chooses an unsafe reversal. Swenson *et al.* showed a permutation family where *k* is *Λ*(*n*) (worst-case for *k*) and, in this case, the algorithm is quadratic. However, tests performed by the authors showed that *k* generally is a constant smaller than 1 and independent of the permutation size. Because of this, the algorithm has, with high probability, execution time O(nlogn)[6].

A more general evolutionary model than the HP model was proposed by Yancopoulos, Attie and Friedberg [7] called the *Double Cut and Join* operation (DCJ). It allows the study of evolutionary scenarios between genomes which are composed by one or more chromosomes, either linear or circular. This universal operation accounts for reversals, translocations, fusions and fissions. Transpositions and block interchanges are modelled by two DCJ operations. Bergeron, Mixtacki and Stoye gave a linear time algorithm to compute the DCJ distance between two genomes [8]. Braga and Stoye studied the solution space of the problem of sorting by DCJ and developed an algorithm to count the number of optimal sorting sequences [9]. Additionally, they demonstrated that any optimal DCJ sorting sequence can be obtained from another one by applying replacements of consecutive operations. However, the problem of finding the shortest number of replacements is still open and an algorithm to enumerate all DCJ rearrangement scenarios is currently not available. Furthermore, the DCJ model appears less relevant then the classical HP model as it allows for mutations that are rarely or never observed in biological data (*e.g.* circular chromosomes for eukaryotes) [10].

### **Enumeration of all solutions to the**SPSR problem

The traditional SPSR algorithms for the HP model however output just one optimal sequence of reversals, while the space of optimal solutions can be huge. Hence, the solution produced by such algorithms may differ from the scenario which really occurred during the evolution of the genome, even when such scenario indeed verifies some optimality criterion. For instance, the permutation (−4,−11,6,−9,−2,1,−8,3,−10,7,−5) has 6345019 optimal solutions.

#### Deterministic approach

Given a permutation *π*^0^ and a target permutation *π*^*d*^, an *optimal 1-sequence* is a reversal that, when applied to *π*^0^, produces a permutation *π*^1^such that *d*(*π*^1^,*π*^*d*^) =* d*(*π*^0^,*π*^*d*^)−1. In the same way, an *optimal i-sequence* is a sequence of *i* reversals that, when applied to *π*^0^, produces a permutation *π*^*i*^such that *d*(*π*^*i*^,*π*^*d*^) =* d*(*π*^0^,*π*^*d*^)−*i*.

In 2003, Siepel proposed an algorithm which calculates the set of all *optimal 1-sequences* of a given permutation in time *O*(*n*^3^) [11]. It is thus easy to see that, by iterating this algorithm, we can obtain the set of all optimal *d*(*π*^0^*π*^*d*^)-sequences that sort the permutation *π*^0^into *π*^*d*^. Recently, Swenson, Badr and Sankoff presented a quadratic algorithm to enumerate the optimal 1-sequences of a permutation [12].

#### Probabilistic approach

York, Durret, and Nielsen proposed in 2002 a Bayesian approach for the problem of inferring the history of inversions which separate the homologous chromosomes from two different species [13]. The method is based on a Markov Chain Monte Carlo (MCMC) approach and models the occurrence of rearrangement events by a Poisson process. Additionally, all possible inversions are supposed to occur with equal probability and the authors do not impose the restriction of parsimonious scenarios to the solutions (*i.e.* they do not require the sorting sequence to have minimum size). This was extended in 2004 to include translocations [14]. In 2003, Mikls proposed an MCMC approach based on a stochastic model of inversions, transpositions and inverted transpositions [15].

The methods of Mikls and York *et al.* were designed to infer the sequence of rearrangement events that explain the difference between two species. Larget *et al.* developed a method to analyse the complete mitochondrial genome rearrangements of 87 metazoa taxa [16,17]. Their approach used an MCMC model to estimate the phylogeny and ancestral genome arrangements considering only reversals. This led to the software BADGER.

An MCMC approach was also proposed by Mikls and Darling in 2009 for sampling parsimonious reversal histories [18]. The method is implemented in the software MC4Inversion. It uniformly samples the set of all optimal paths and can estimate the total number of optimal sorting paths.

### Traces

Bergeron *et al.* introduced the concept of *traces* for the SPSR problem. This concept allows the organisation of a set of optimal solutions into classes [19]. If sequences of reversals are identified as words, Bergeron *et al.* define a relation over them: if *ρ* and *θ* are reversals (intervals) and they show no overlap, then the words *ρθ *and *θρ* are said to be equivalent. We say that *ρ *and *θ*commute. Based on this relation, any word that contains the subword *ρθ* is equivalent to the same word obtained by replacing *ρθ *by *θρ*. For example, the sequences of reversals (words) {1}{1,2,3}{2,3,4} and {1,2,3}{1}{2,3,4} are equivalent because the reversals {1} and {1,2,3} commute. Inversely, none of these sequences of reversals is equivalent to {1}{2,3,4}{1,2,3} because the reversals {1,2,3} and {2,3,4} overlap.

A class of optimal reversal sequences over this relation is called a *trace*. Bergeron *et al.* proposed that for a given signed permutation *π*, the set of all optimal solutions is a union of traces. Thus, traces can be used to produce a more relevant result to the SPSR problem because they provide a more compact representation of an enormous set of solutions.

#### Normal form of a trace

An element *s* of a trace *T* is in its *normal form* if it can be decomposed into subwords *s *=* u*_1_|…|*u*_*m *_such that: 

• every pair of elements of a subword uicommute;

• for every element ρof a subword ui(i > 1), there is at least one element θ of the subword ui−1such that ρand θ do not commute;

• every subword uiis a nonempty increasing word under the lexicographic order.

A theorem by Cartier and Foata states that, for any trace, there is a unique element that is in normal form [20]. This allows the representation of traces through their normal forms.

The number of subwords in a trace denotes its *height*. The *size* of a trace *T* is the number of solutions which it represents. The trace {1,2,4}{3}|{1,3,4}|{2,3,4} has height 3 and size 4 because it represents just the solutions: {3}{1,2,4}{1,3,4}{2,3,4}, {1,2,4}{3}{1,3,4}{2,3,4}, {1,2,4}{1,3,4}{3}{2,3,4}, and {1,2,4}{1,3,4}{2,3,4}{3}.

A trace *T* that contains an optimal sequence *ρ*_1_…*ρ*_*d *_can be represented by a partial ordering of the set $P_{T}=\{\rho_{1},\ldots ,\rho_{d}\}$. We say that *ρ*_*i *_<_* T*_*ρ*_*j*_ if and only if *ρ*_*i*_ is always before *ρ*_*j*_ in the elements of *T*. For example, in the trace *T *= {1,2,4}{3}|{1,3}|{3,4}, the elements of $P_{T}$ are {1,2,4}, {3}, {1,3}, and {3,4}, and the relations are {1,2,4} <_*T *_{1,3}, {1,3}<_*T *_{3,4}, and {1,2,4}<_*T *_{3,4}. Notice that the reversal {3} is not comparable to the others by the relation <_*T*_ because, given that it does not overlap the other reversals, it can be placed in the sequence before or after any of them.

The set $P_{T}$ and the relation <_*T *_form a partially ordered set (*poset*). The *width* of a trace (or poset) is a maximum cardinality set of elements of $P_{T}$ that are not comparable by the relation <_*T*_. It is at least (but in general not equal to) the maximum size of a subsequence *u*_*i*_ in the normal form of a trace.

#### Tree representation of a set of solution traces

**Figure 1:**
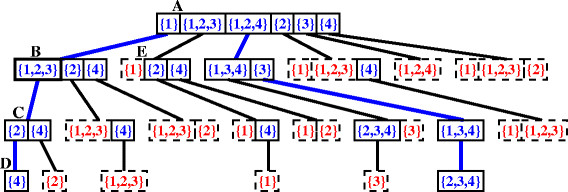
**Tree representation of the solution traces of the permutation *π* =(−3,2,1,−4).** In this representation, only the values inside of solid boxes are reversals that effectively are in the structure. The values inside of dashed boxes are reversals which are optimal 1-sequences but, when combined with their parent trace, lead to traces that were inserted in another branch of the tree. The wide edges show the two traces which sort the permutation: {1}{1,2,3}{2}{4} and {1,2,4}{3}|{1,3,4}|{2,3,4}.

A set of solution traces can be represented using a sorted tree similar to the one shown in Figure 1 (that records the traces that sort the permutation *π*^0^ = (−3,2,1,−4) into *ι*_4_). A node of the tree represents a set of reversals that is sorted in lexicographic order. The root node contains the optimal 1-sequences of the original permutation. To each reversal *ρ* of a non-leaf node is attached a subtree which groups the reversals that are lexicographically bigger than it or that should be applied after it. For example, in Figure 1, node *A* contains the optimal 1-sequences of *π*^0^ and the reversal {1} of node *A* has a subtree, rooted at node *B*, attached to it. Node *B* contains the optimal 1-sequences of the permutation *π*^1^ obtained after applying the reversal {1} over *π*^0^.

Every path from the root to a node at level *i* of the tree gives us an *i*-trace. An *i*-trace represents a set of solutions which sort *π*^*k*^ into *π*^(*k* + *i*)^by using the same set of *i* reversals, respecting the overlap relationship among them. If *i *=* d*(*π*^0^,*π*^*d*^), then we have a trace that sorts the permutation *π*^0^ into *π*^*d*^.

### **Complete enumeration of solution traces for the**SPSR problem

Braga *et al.* combined Siepel’s algorithm with the concept of traces [21] and developed an algorithm for enumerating all solution traces of a given permutation. As a single trace can represent a big number of solutions, by enumerating traces we can generate a set much smaller than the complete set of solutions. Moreover, the clustering of solutions provided by the traces offers to the biologist a better information on the characteristics of the blocks of elements of the permutation which are being affected by the reversals.

Considering some biological criteria, constraints can be applied in the selection of the reversals during the process of enumeration. Thus, the size of the output can be reduced [21,22]. For example, common intervals between two permutations can be used to model clusters of co-localised genes. These clusters are intervals of the genomes which are composed by the same genes but not necessarily in the same order and orientations. In this context, we can determine a biological constraint that forbids or imposes a maximum number of reversals that break this type of intervals.

The algorithm proposed by Braga *et al.* explores the tree of solution traces in breadth-first manner and adopts a complex data structure to keep the intermediary information into the main memory and disk [23]. Due to the strategy adopted in this algorithm, in this text we shall refer to it as the *Breadth-First Algorithm* or BFA for short.

In a previous work, instead of exploring the universe of solutions in breadth-first manner, we adopted a depth-first strategy to explore the branches of the tree of solution traces [24]. This *Depth-First Algorithm* (or DFA for short) makes use of a stack structure to keep the intermediary data on the main memory only. With this solution, we greatly reduced the amount of data which must be kept in the main memory and we eliminated the disk accesses. However, this algorithm cannot be used with most of the biological constraints developed by Braga *et al.*, and cannot compute the total number of solutions that is represented by the set of traces.

The time complexity of the BFA algorithm is $O(Nn^{k_{\max}+4})$, where *k*_*max*_ is the maximum width of a trace and *N* is the number of traces of solutions [21]. The complexity of the DFA algorithm is *O*(*Nn*^4^2^*n*^) [25].

Recently, Badr, Swenson and Sankoff adapted the two algorithms of trace enumeration [25]. The strategy consists in grouping *i*-traces according to the permutation that they produce when their sequences of reversals are applied to the original permutation. As many traces can produce the same intermediary permutation, by grouping them, the authors avoid unnecessary computations. Instead of generating the set of optimal 1-sequences for every *i*-trace, they compute this set just for the intermediary permutation which groups a set of *i*-traces. Despite the gain of 70% over the execution time of the BFA algorithm and 50% over the DFA algorithm, the methods proposed by Badr, Swenson and Sankoff use a considerable amount of the main memory to keep the groups of *i*-traces and permutations.

### **Partial enumeration of solution traces for the**SPSR problem

Although sets of traces are smaller than their equivalent sets of solutions, the number of traces also increases exponentially with the size of the permutations and their reversal distance. Thus, for big permutations (*n*≥15), the time necessary to produce the complete set of traces makes impracticable any analysis.

For big permutations, instead of enumerating the complete set of traces, we could study alternative evolutionary scenarios by producing a sampling of this set. We call this sampling a “*Partial Enumeration of Traces*” and, in this work, we propose three new algorithms for doing this sampling.

#### New algorithms

The three algorithms were designed to enumerate traces while a given execution time limit is not reached. The first (RA) is very simple and constructs the traces through a random selection of reversals in the list of optimal 1-sequences. The second algorithm (DFALT) represents a slight modification of the DFA algorithm. Finally, the last algorithm (SWA) is more elaborate and makes uses of a sliding window strategy to improve the enumeration of traces.

We implemented the proposed algorithms and tested them with sets of random permutations. While processing small permutations, the DFALT algorithm is able to sample a number of traces higher than the ones obtained by the other solutions. However, as the size of the permutations increases, the algorithm SWA outperforms the others with respect to the number of enumerated traces.

#### Quality of sampling

To demonstrate that the sampling is from the uniform distribution and to determine the time that is necessary to obtain a good sampling are not easy tasks. Indeed, it has been conjectured that this is *♯*P-complete [10]. It may however be enough in some cases to show that the sampling preserves in practice a characteristic that is biologically relevant. One such characteristic is the average length of the reversals in optimal scenarios. Indeed, the literature contains studies of genomes that appear subjected to reversals of mainly small or intermediate sizes [26-29]. In this context, sampling traces whose average reversal length follows a distribution statistically similar to the distribution observed for the complete set of traces can be important to validate or invalidate an a posteriori biological interpretation. We could qualitatively show that when we increase the execution time, the DFALT and SWA algorithms obtain sets whose distributions of traces do tend to approach the distributions observed for the complete set of traces as concerns the average reversal length of the traces, and also the height.

## Methods

### State of Art – Algorithms for traces enumeration

Before introducing the algorithms for partial enumeration of traces, in this section we make a quick presentation of the algorithms which were designed for the enumeration of the complete set of solution traces.

#### **Breadth-first algorithm –**BFA

Braga *et al.* proposed the first algorithm for enumeration of solution traces [21]. This explores the tree of solution traces in a breadth-first manner.

First, the algorithm lists the set of optimal 1-sequences of the original permutation *π*^0^. These optimal 1-sequences are equivalent to a list of 1-traces of the permutation *π*^0^.

Then at each iteration *i*(1 <* i *≤* d*(*π*^0^,*π*^*d*^)), the algorithm applies each (*i*−1)-trace *t* on *π*^0^ to produce a new permutation *π*^(*i*−1)^ (i.e. *π*^(*i*−1)^ =* π*^0^∘*t*). The list of optimal 1-sequences of *π*^(*i*−1)^ is thus obtained, and each reversal of this list is added to the (*i*−1)-trace *t* to generate a new set of *i*-traces.

When the algorithm finishes to process level *i*=*d*(*π*^0^,*π*^*d*^), all traces which sort *π*^0^into *π*^*d*^have been enumerated.

#### **Depth-first algorithm –**DFA

A depth-first strategy was adopted by Baudet and Dias to explore the tree of solution traces [24].

The algorithm makes use of a stack structure to handle the data produced during the process. Each level of the stack has a list of reversals sorted in lexicographic order. Additionally, the sequence of reversals constructed with the first reversal of each level, from the bottom to the top of the stack, represents the current *i*-trace.

First, the list of optimal 1-sequences of the permutation *π*^0^ is pushed into the first level of the stack. While the stack is not empty, the algorithm gets the current *i*-trace *t* and applies it to the permutation *π*^0^ to produce the permutation *π*^*i*^. Each reversal *ρ* of the list of optimal 1-sequences of *π*^*i *^is added to the list that will be pushed into the top of the stack only when it is the last reversal of the (*i* + 1)-trace *t*^*″*^=*t* + *ρ*. If the reversal *ρ* does not appear in the last position of the (*i* + 1)-trace *t*^*″*^, it means that it belongs to another branch of the tree of solution traces and, therefore, it can be ignored.

When the stack reaches the level *i *=* d*(*π*^0^,*π*^*d*^), the algorithm outputs the current trace *t* and removes from the list the reversal that is on the top of the stack. Every time the top of the stack contains an empty list, the algorithm pops it and removes the first reversal of the list that is in the new top. The algorithm finishes when the stack becomes empty.

#### BFA**and**DFA**with permutation grouping**

During the enumeration of traces which sort *π*^0^into *π*^*d*^, different *i*-traces can sort *π*^0^ into the same intermediary permutation *π*^*i*^. Based on this observation, Badr, Swenson and Sankoff adapted the BFA and DFA algorithms to speed-up the trace enumeration [25].

The strategy consists in grouping *i*-traces according to the permutation that they produce when their sequences of reversals are applied to the original permutation. Instead of computing sets of optimal 1-sequences for every *i*-trace, this procedure is performed only for each intermediary permutation which appears on the level *i*.

Tests performed by the authors show that, on average, this change in the algorithms results in a gain of 70% and 50%, respectively, over the total execution time of the algorithms BFA and DFA.

### New algorithms – Partial enumeration of traces

Due to the exponential nature of the set of solutions, the algorithms that sort all solution traces are not suited for processing big permutations.

With the objective of calculating alternative evolutionary scenarios for big permutations, we developed three different algorithms that perform a partial enumeration of the complete set of solution traces of a given permutation.

The proposed algorithms output traces while they do not reach the stopping criterion which, in our case, is a given execution time limit.

#### **Random algorithm –**RA

A very simple solution for the partial enumeration of traces is to construct random traces.

Let *π*^0^ be the original permutation and *π*^*d *^the target permutation. This method consists in generating a trace through the random selection of a reversal among those in the set of optimal 1-sequences of each permutation *π*^*i*^ which is between *π*^0^and *π*^*d*^(0 ≤* i *<* d *=* d*(*π*^0^,*π*^*d*^)). This process is repeated while the execution time limit is not reached.

#### **Depth-first algorithm limited by time –**DFALT

The algorithm DFA explores the tree of solution traces branch by branch. Moreover, every time it reaches a leaf node, it outputs a new trace. Consequently, another simple alternative to producing a set of traces is to use the algorithm DFA and introduce a verification over the elapsed time to interrupt its execution when the time limit is reached.

Observe that the same procedure cannot be adapted to the algorithm BFA. As it outputs the enumerated traces only when it reaches the last level, the necessary time limit to output at least one trace would be very close to the time required to enumerate all traces.

#### **Sliding window algorithm –**SWA

Let *π*^*k*^ be an intermediary permutation that is obtained after applying the first *k* reversals of an optimal sequence of reversals which transforms *π*^0^into *π*^*d*^(1 ≤* k *<* d *=* d*(*π*^0^,*π*^*d*^)). In this context, we can define the *k*-trace *X* and the *l*-trace *Y *, where *l *=* d*−*k*. *X* and *Y * are, respectively, the traces which represent all solutions that transform *π*^0^ into *π*^*k*^ and, *π*^*k*^ into *π*^*d*^.

**Figure 2:**
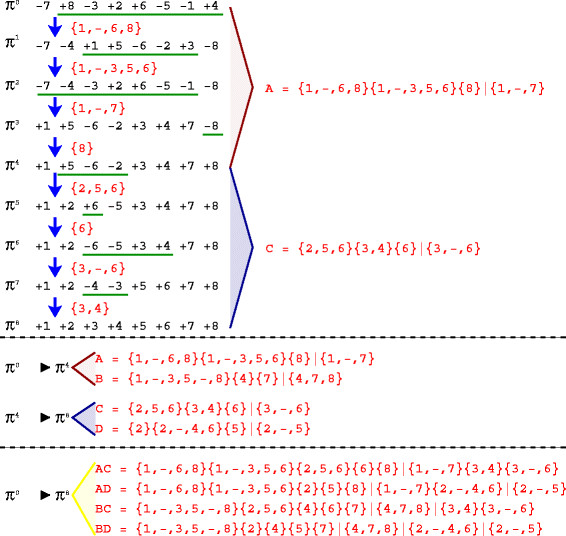
**Building a 8-trace with 4-traces.** This schema shows a sequence of reversals that optimally sorts *π*^0^ = (−7, + 8,−3, + 2, + 6,−5,−1, + 4) into *π*^8^ = ( + 1, + 2, + 3, + 4, + 5, + 6, + 7, + 8). This sequence of reversals is represented by the 8-trace {1,−,6,8}{1,−,3,5,6}{2,5,6}{6}{8}|{1,−,7}{3,4}|{3,−,6}. This 8-trace can be obtained by the application of the reversals of the 4-trace *C *= {2,5,6}{3,4}{6}|{3,−,6} (it sorts *π*^4^into *π*^8^) to the sequence of reversals of the 4-trace *A *= {1,−,6,8}{1,−,3,5,6}{8}|{1,−,7} (it sorts *π*^0^into *π*^4^). The algorithm DFA can be used to obtain the two 4-traces which sort *π*^0^into *π*^4^and the two 4-traces which sort *π*^4^into *π*^8^. Combining these 4-traces, we obtain all 8-traces of solutions which sort *π*^0^into *π*^8^and have *π*^4^as an intermediary permutation.

If we get the reversals of *Y * and add each one of them, sequentially, to *X*, we produce a trace *Z* that transforms *π*^0^ into *π*^*d*^. For example, Figure 2 shows a sequence of reversals which optimally sorts the permutation *π*^0^ = (−7, + 8,−3, + 3, + 6,−5,−1, + 4) into the permutation *π*^8^ = ( + 1, + 2, + 3, + 4, + 5, + 6, + 7, + 8). The 4-trace *A *= {1,−,6,8}{1,−,3,5,6}{8}|{1,−,7} represents a solution trace which transforms *π*^0^ into the intermediary permutation *π*^4^. In the same way, the 4-trace *C *= {2,5,6}{3,4}{6}|{3,−,6} represents a solution trace which transforms *π*^4^ into *π*^8^. By adding each reversal of *C* into the trace *A*, we build the 8-trace *AC *= {1,−,6,8}{1,−,3,5,6}{2,5,6}{6}{8}|{1,−,7}{3,4}|{3,−,6} which sorts *π*^0^into *π*^8^.

This strategy of combining small traces to construct a bigger one can be used in a sliding window algorithm. The set of all intermediary permutations which is produced by an optimal sequence of reversals is processed by a window of size *w* that produces sets of *k*-traces (1 ≤* k *≤* w*) which transform: *π*^0^ into *π*^1^, *π*^0^ into *π*^2^, …, *π*^0^ into *π*^(*w*−1)^, *π*^0^ into *π*^*w*^, *π*^1^ into *π*^(*w* + 1)^, *π*^2^ into *π*^(*w* + 2)^, …, *π*^(*d*−*w*)^ into *π*^*d*^, *π*^(*d* + 1−*w*)^ into *π*^*d*^, …, *π*^(*d*−1)^ into *π*^*d*^.

After that, these sets of *k*-traces (1 ≤* k *≤* w*) can be combined in the following way: 

• 1-traces that transform π0 into π1 with w-traces that transform π1 into π(w + 1) to generate (w + 1)-traces which transform π0 into π(w + 1);

• 2-traces that transform π0 into π2 with w-traces that transform π2 into π(w + 2) to generate (w + 2)-traces which transform π0 into π(w + 2);

• …

• (d−1)-traces that transform π0 into π(d−1)with 1-traces that transform π(d−1)into πdto generate d-traces which transform π0into πd.

The first step of this algorithm consists in generating a random set of intermediary permutations. To do this, we can adapt the algorithm RA to return the list of all intermediary permutations (*π*^*i*^, 0 ≤* i *≤* d*).

To produce the set of all *k*-traces (1 ≤* k *≤* w*) that transform *π*^*i *^into *π*^(*i* + *k*)^, we can use the algorithm DFA.

In our example of Figure 2, the algorithm DFA would output the 4-traces *A *= {1,−,6,8}{1,−,3,5,6}{8}|{1,−,7} and *B *= {1,−,3,5,−,8}{4}{7}|{4,7,8}, which transform *π*^0^ into *π*^4^, and the 4-traces *C *= {2,5,6}{3,4}{6}|{3,−,6} and *D *= {2}{2,−,4,6}{5}|{2,−,5}, which transform *π*^4^ into *π*^8^. With the combination of these 4-traces, we can obtain four 8-traces which transform *π*^0^ into *π*^8^ passing by the intermediary permutation *π*^4^(*AC*,*AD*,*BC*, and *BD*).

### Tests

The algorithm BFA was implemented in Java by Braga [30]. Starting from this Java source code, we implemented the algorithm DFA in order to adopt the same Java Objects that were used by Braga. The algorithms RADFALT, and SWA were also implemented under the same Java package structure.

The tests were performed on an Intel Pentium 4 HT 3.0 GHz with 2.0 GB of RAM running Ubuntu. To avoid the influence of swap operations on the performance of the structures, we limited the maximum memory that the Java Virtual Machine could allocate to 1.0 GB (parameter -Xmx1024m).

During the tests we collected the maximum amount of main memory used by the algorithms. The memory was measured through a separated thread that at regular intervals collected the memory used by the Java Virtual Machine (Object Runtime: methods totalMemory() and freeMemory()).

Random permutations were generated to test the algorithms. Since the package implemented by Braga does not work with permutations that have hurdles, the generated permutations should have no hurdles. The decision to ignore hurdles is based on the very small probability of finding them in random permutations [31].

Given a number *n* of elements and a reversal distance *d*, starting from the identity permutation $\iota_{n}=\pi^{d}$, a random permutation is generated through the application of successive reversals in the following way: while there is more than one adjacency (pair of elements which appear together with the same relative orientations in the current and target permutations) in the permutation *π*^*i*^, two adjacencies are chosen at random and their positions are used to define the reversal *ρ*_*i*_; otherwise, two positions of the permutation are chosen at random to define *ρ*_*i*_. After applying *ρ*_*i *_to *π*^*i*^ to obtain *π*^(*i*−1)^, we verify if either *d*(*π*^(*i*−1)^,*π*^*d*^)<*d*(*π*^*i*^,*π*^*d*^) or *π*^(*i*−1)^ contains hurdles. If one of these conditions are observed, we discard *π*^(*i*−1)^ and generate a new random reversal *ρ*_*i*_ to apply to *π*^*i*^. The process finishes when we produce the permutation *π*^0^.

## Results and discussions

### Evaluating the time necessary to enumerate all traces

Before performing comparative tests with the three proposed algorithms (RA, DFALT, and SWA), we evaluated the average time which is necessary to enumerate all traces of a given permutation. The objective here is to show the exponential behaviour of the sizes of the sets of traces, depending on the number of elements of the permutations and their reversal distances, and to collect information to determine the time limits that will be used to evaluate the proposed algorithms. We shall see later how to choose such a time limit in general, or some other stop criterion.

**Figure 3:**
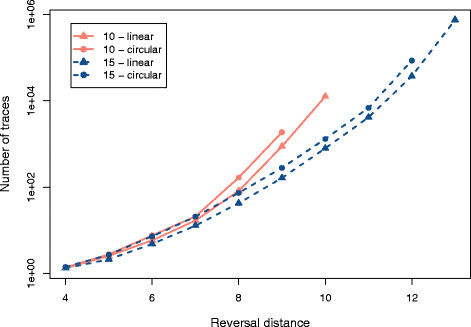
**Average number of traces.** Sets of 500 random permutations (linear and circular) with 10 and 15 elements and different reversal distances were processed with the algorithm DFA. For each set, we calculated the average number of traces.

**Figure 4:**
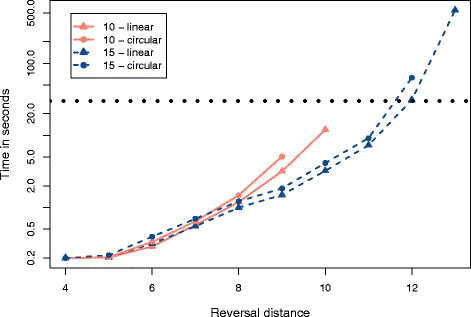
**Average execution time.** Sets of 500 random permutations (linear and circular) with 10 and 15 elements and different reversal distances were processed with the DFA algorithm. For each set, we calculated the average execution time. The horizontal dotted line indicates 30 seconds.

We created sets of 500 random linear and circular permutations with, respectively, 10 and 15 elements with reversal distances between 4 and 13. These values were chosen because they allow the total enumeration of traces, for the set of 500 permutations, in a reasonable time. For example, just the set (*n *= 15, *d*(*π*) = 13) required three days to be processed. For each permutation, we enumerated all traces with the algorithm DFA, and we collected the execution time. For each set of permutations, we calculated the average number of traces and the average execution time. Figures 3 and 4 show the plots which were produced with the collected values.

The algorithm DFA was chosen because it is faster than the algorithm BFA. We could also adopt the algorithm DFA with a permutation grouping that reduces the total execution time by 50%. However, the permutation grouping routine requires a big amount of main memory to keep the associations between intermediary permutations and *i*-traces. Additionally, the results of the work of Badr, Swenson and Sankoff (Figure 3[25]) show that the adapted version of the algorithm DFA outperforms the basic version only when the permutations have reversal distance bigger than 8. As our testing environment had a limited amount of main memory, we opted to use instead the basic version of the algorithm DFA.

Figure 3 shows that the number of traces grows exponentially with the number of elements and with the reversal distance. For a same reversal distance, we can see that the number of traces grows with the ratio *d*(*π*)/*n*. For example, when we fix the value 9 for the reversal distance, permutations with 10 elements have on average more traces than permutations with 15 elements. The same observations made for Figure 3 can be applied to Figure 4, and this means that the time is proportional to the number of traces which must be enumerated.

The average amount of traces observed for circular permutations is bigger than the one observed for linear permutations with the same reversal distance. For every reversal in a linear permutation, there exists two equivalent reversals in the corresponding circular permutation. Thus, circular permutations have a higher number of optimal solutions than linear permutations. This characteristic is indicated by the curves of Figure 3.

### Number of enumerated traces versus execution time

To evaluate the proposed algorithms, we decided to adopt a set of permutations which lead to an average execution time that is neither too short, nor too long. As the behaviour of linear and circular permutations are similar, we opted for performing tests only with linear permutations. Based on these criteria, we chose the set (*n *= 15, *d*(*π*) = 12) which leads to an average execution time of, approximately, 30 seconds.

We processed the selected set of permutations with the algorithms RA, DFALT, and SWA. In the case of the algorithm SWA, we adopted the values 4, 5 and 6 for the parameter window size. These values were chosen with the aim of obtaining a compromise between the number of enumerated *w*-traces and the time lost with the dead branches. To facilitate the description along the text, we shall refer to these algorithms as, respectively, SWA4, SWA5, and SWA6.

First, for each permutation *P*, we got the set of all its traces and we counted the number of traces that have height *H*(2 ≤* H *≤ 12). We also counted the number of traces that have average reversal length *R*(2≤*R*≤11).

Considering that the average time to process the permutations of the selected set is 30 seconds, we used the algorithms with the following time limits: 6, 12, 18, 24, 30, and 36 seconds. Each permutation *P* was processed by each pair (algorithm *A*, time limit *T*). For each of these executions, we calculated the percentage of all traces of height *H* (resp., average reversal length *R*) of the permutation *P* that we sampled with the algorithm *A* within the time limit *T*.

**Figure 5:**
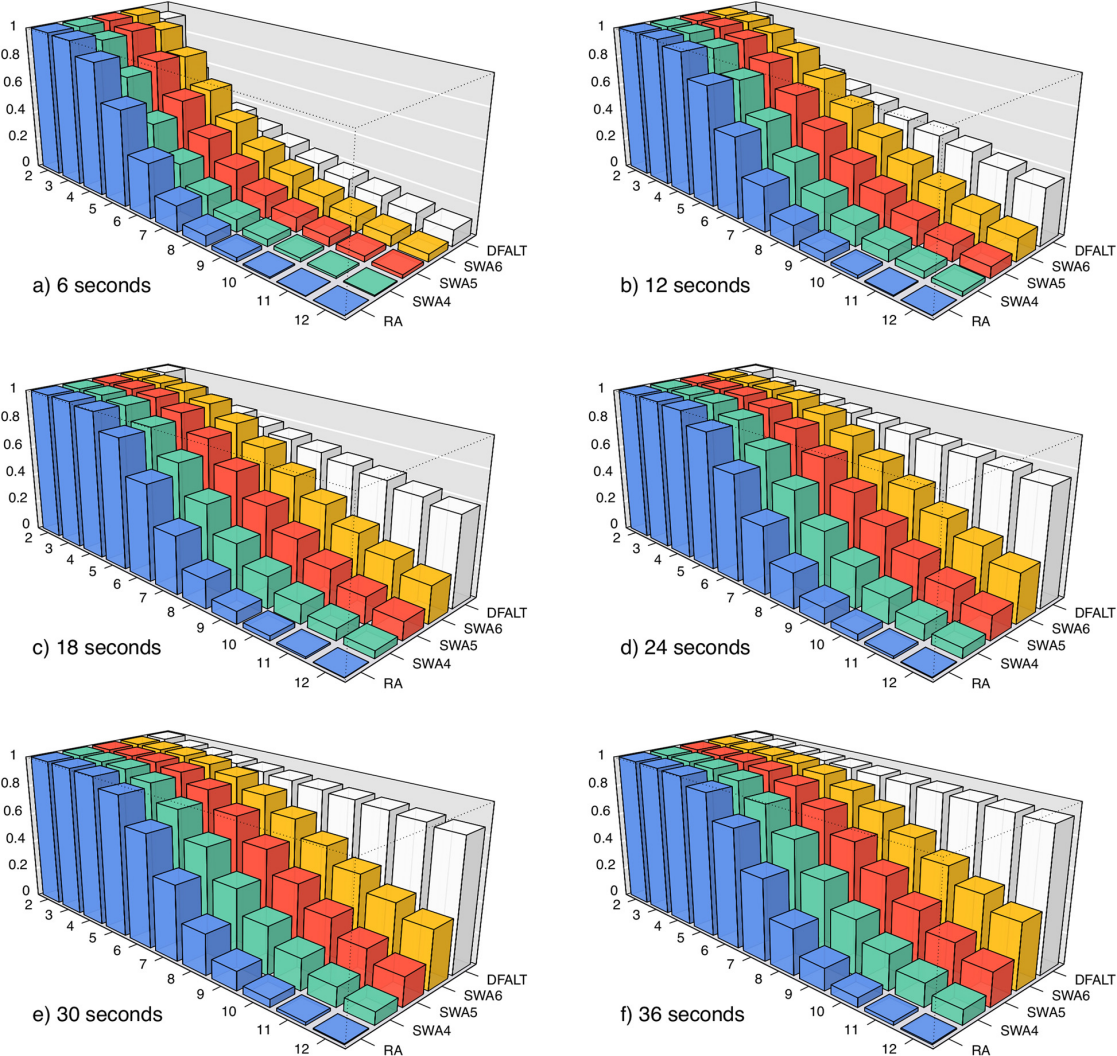
**Average percentage of traces with height *H* which were calculated by each algorithm.** A set of 500 random permutations with *n *= 15 and *d*(*π*) = 12 were processed by RA, DFALT, SWA4, SWA5, and SWA6. The following time limits were imposed to the algorithms: 6, 12, 18, 24, 30, and 36 seconds. For each triplet (*A*, *H*, *T*), we calculated the average percentage of traces with height *H* shown by the 500 permutations in the execution of algorithm *A* inside of the time limit *T*. In each plot, the axes *x*, *y*, and *z* represent, respectively, the heights, the algorithms and, the average percentage values.

**Figure 6:**
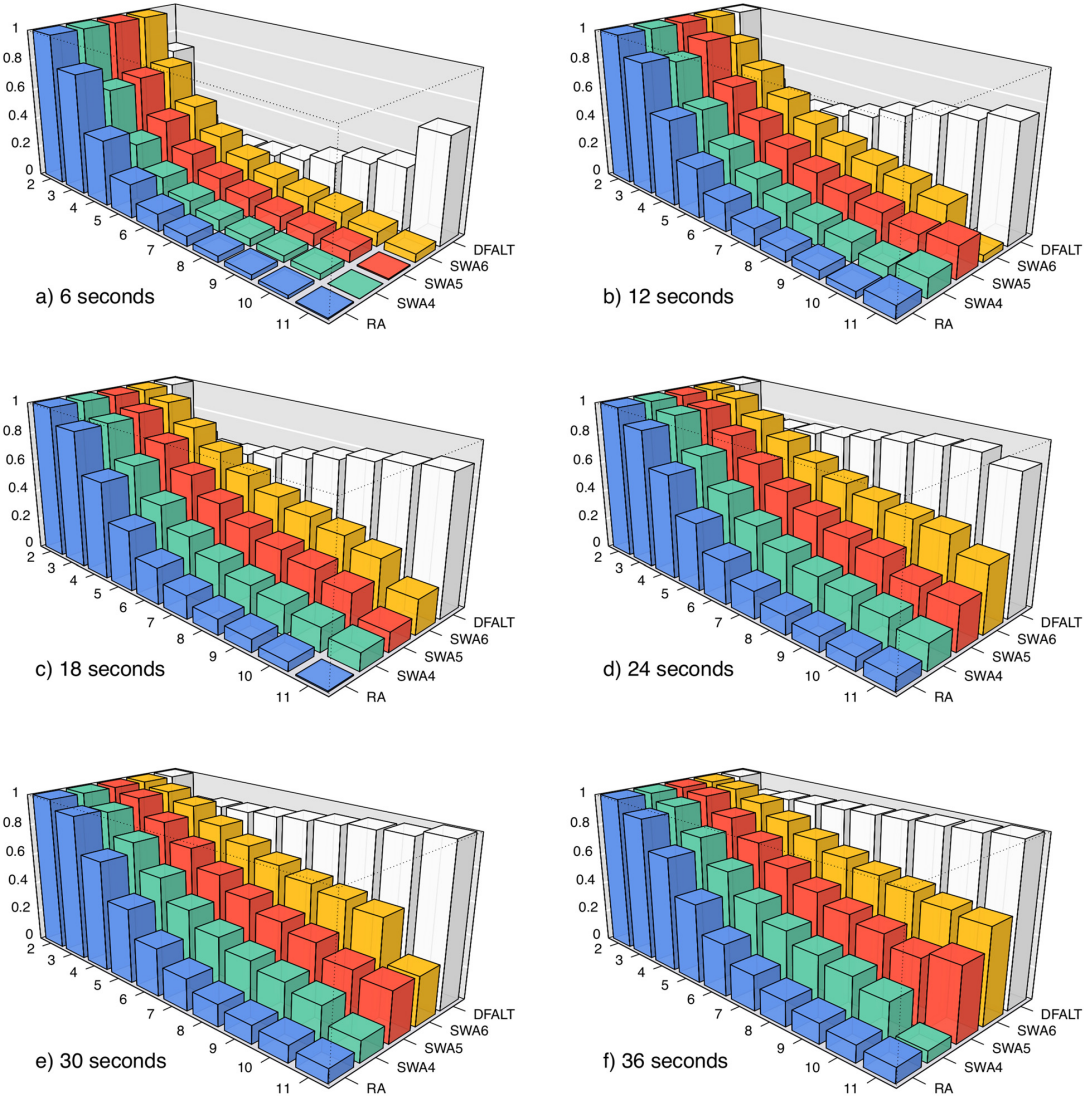
**Average percentage of traces with average reversal length *R* which were calculated by each algorithm.** A set of 500 random permutations with *n *= 15 and *d*(*π*) = 12 were processed by RA, DFALT, SWA4, SWA5, and SWA6. The following time limits were imposed to the algorithms: 6, 12, 18, 24, 30, and 36 seconds. For each triplet (*A*, *R*, *T*), we calculated the average percentage of traces with average reversal length *R* shown by the 500 permutations in the execution of algorithm *A* inside of the time limit *T*. In each plot, the axes *x*, *y*, and *z* represent, respectively, the reversal lengths, the algorithms and, the average percentage values.

Finally, for each pair (*A*, *T*), we calculated for the set of 500 random permutations the average percentage of all traces of height *H* (average reversal length *R*) that were enumerated by *A* within the time limit *T*. The plots in Figures 5 and 6 show the collected data for the parameters *H* and *R* respectively.

Among the proposed solutions, the algorithm DFALT is the only one which is deterministic. Figures 5 and 6 show that the increment of the execution time corresponds to a gradual increase in the number of enumerated traces.

For the non-deterministic algorithms, Figures 5 and 6 show also a gradual increment in the number of enumerated traces. However, these algorithms do not present a good capacity for sampling traces that have high height or high average reversal length. We can see that the algorithm RA has the worst results and the algorithm SWA6 has the best results among the non-deterministic algorithms. Notice however that, as we shall see later, since the number of traces with high height or high average reversal length are rare in general, this will not affect much the observed distributions of such parameters for the partial enumeration of traces relatively to a full enumeration.

The lower the height of a trace, the higher is the number of solutions that it represents. This happens because, when we have a small number of overlaps among the reversals, we have a higher number of possible combinations for the sequence of reversals. The same observation can be made for traces that have a small value for the average of the reversal length. When the reversals have small size, the probability of overlap among them decreases and, consequently, the number of solutions that can be represented by the traces increases. Thus, randomly, we have a bigger chance of producing a trace with low height or low average reversal length. This explains the behaviour shown by the non-deterministic algorithms in Figures 5 and 6.

### Processing big permutations

The average time to process this set of permutations (*n *= 15, *d *= 12) is just 30 seconds. It is a set of permutations whose traces can be easily enumerated. Nonetheless, these algorithms were developed with the objective of enumerating traces of big permutations which demand a huge processing time.

**Figure 7:**
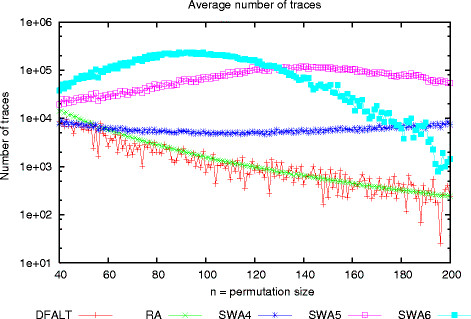
**Average number of traces produced by each algorithm in 60 seconds.** Sets of 100 random permutations with 40 ≤* n *≤ 200 and *d*(*π*) = ⌈(*n* + 1)/2⌉ were generated and processed with the algorithms RA, DFALT, SWA4, SWA5, and SWA6 during a time limit of 60 seconds. The plot shows the average number of traces produced by each algorithm for each set of permutations.

**Figure 8:**
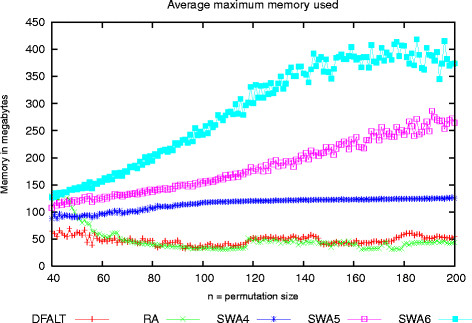
**Maximum amount of main memory used by the partial enumeration algorithms.** Sets of 100 random permutations with 40 ≤* n *≤ 200 and *d*(*π*) = ⌈(*n* + 1)/2⌉ were generated and processed with the algorithms RA, DFALT, SWA4, SWA5, and SWA6 during a time limit of 60 seconds. The plot shows the average maximum amount of main memory used by the algorithms while processing each set of 100 permutations.

To check whether they were capable of doing this, we created sets of 100 random permutations with a number of elements varying between 40 and 200 and a reversal distance *d *= ⌈(*n* + 1)/2⌉. Each permutation was processed by each proposed algorithm with a time limit of 60 seconds. For each execution, we collected the number of enumerated traces and the maximum amount of memory used by the algorithm. Figures 7 and 8 show, for each algorithm and for each value of *n* respectively, the average number of enumerated traces and the average memory usage observed during the executions of each set of 100 permutations.

We can see in Figure 7 that the number of traces that are enumerated by DFALT decreases as the size of the permutations increases. This phenomenon is associated with the time that this algorithm spends processing dead branches in the tree of traces.

RA has a curve very similar to the one shown by DFALT but its shape has a different explanation. When we increase the number of elements of the permutation and, consequently, the initial reversal distance, we have that the time that is spent on the analyses of the breakpoint graph to find an optimal 1-sequence also grows. Because of this, the number of traces which are enumerated by the algorithm RA decreases when we increase the initial reversal distance of the permutations.

The reason that makes the algorithm RA lose in performance does not affect the SWA algorithm. Even with an increment of the initial reversal distance, SWA is all the time concerned with the enumeration of *k*-traces (1 ≤* k *≤* w*). As *w* is usually small, SWA does not lose in performance when producing optimal 1-sequences.

Another advantage of the sliding window strategy is that it produces all *k*-traces that transform *π*^*i*^ into ^*π*(*i* + *k*)^. Because of this, we profit from all the structures that are created for the generation of the optimal 1-sequences. In the case of the algorithm RA, even if we avoid to generate all structures, the created ones are partially explored because just one reversal is considered for each intermediary permutation.

Figure 7 shows that the algorithm SWA is able to enumerate more traces than the other two algorithms when the same time limit is imposed. For permutations with up to 120 elements, the algorithm SWA6 enumerates the highest number of traces. For bigger permutations, the algorithm SWA5 outperforms the algorithm SWA6.

We can see in Figure 7 that the algorithms SWA5 and SWA6 present curves that have a parabolic shape. The number of enumerated traces grows up to a given point, and then starts to decrease. The explanation for this behaviour lies in the process of combining the *i*-traces and the *k*-traces. When we combine *xi*-traces with *yk*-traces, we can create up to *x*×*y*(*i* + *k*)-traces (some of the generated traces can appear more than once). Thus, if the reversal distance of the original permutation increases, the number of combinations (*i*-traces + *k*-traces) and the time that is spent on them also increases.

Generally, a set of 6-traces is bigger than a set of 5-traces and much bigger than a set of 4-traces. As a consequence, we can see that the algorithm SWA6 initially enumerates many more traces but the reduction in the performance also starts earlier than for the other two tested values of window.

Figure 8 shows that RA and DFALT have a small variation in the average memory and that the algorithm SWA consumes more memory. While SWA4 has a more stable memory usage, SWA5 and SWA6 have an ascending curve of memory usage.

While the random algorithms can eventually produce the same trace more than once, DFALT outputs every trace just once. Because of this, when using DFALT, we can print the traces avoiding to keep them in memory with the purpose of controlling duplicated traces.

The higher memory usage of RA is related to the interval where it outputs more traces. When the number of enumerated traces decreases, the amount of space that we need to keep the traces in memory also diminishes. As a consequence, memory consumption reaches a level that is low enough for the maintenance of the objects which are being used to produce the enumeration.

In the case of the algorithm SWA, we have to keep in memory the sets of traces which were enumerated and the set of traces which are going to be combined. To reduce memory consumption, we could print all enumerated traces but, as a result of this, we must add a post-processing step to eliminate the duplicate traces.

### Evaluating the quality of the sampling

When we perform a sampling of a big set of elements, usually we must verify whether the result is unbiased. This implies checking if the output of the algorithms covers the space of solutions uniformly. This task has been conjectured to be *♯*P-complete [10]. We therefore addressed this issue in a different way, and tried instead to show that the sampling strategies developed preserved in practice some important characteristic of the set of all optimal solutions. The characteristics in this case are the average reversal length of the traces, and also the height.

We considered the set of 500 random permutations with *n *= 15 and *d *= 12 and, for each permutation *P*, we calculated the ratios (*number of enumerated traces with height H / total number of enumerated traces of P*) and (*number of enumerated traces with average reversal length R / total number of enumerated traces of P*), using the complete set of traces (Total) and the outputs of the executions of each algorithm. For each permutation, we thus have the distribution of its traces according to the height, and to the average reversal length of the traces.

**Figure 9:**
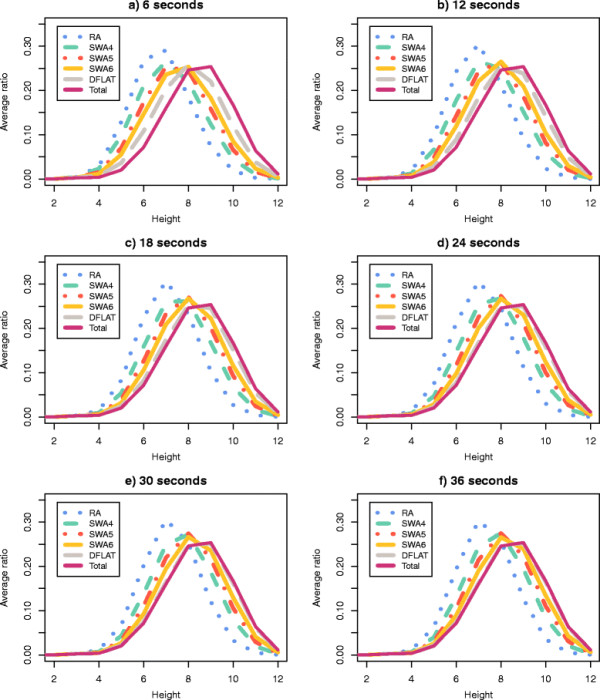
**Average ratio distribution of the traces according to their heights.** Using the set of 500 random permutations with *n *= 15 and *d*(*π*) = 12, we calculated the average ratio distribution, according to the trace height, of the complete set of traces. This procedure was repeated for each pair (algorithm *A*, time limit *T*).

**Figure 10:**
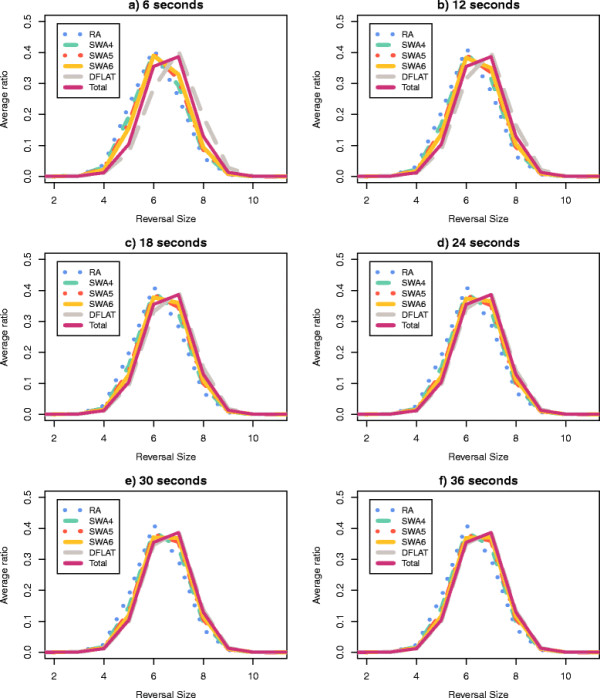
**Average ratio distribution of the traces according to their average reversal length.** Using the set of 500 random permutations with *n *= 15 and *d*(*π*) = 12, we calculated the average ratio distribution, according to the average reversal length, of the complete set of traces. This procedure was repeated for each pair (algorithm *A*, time limit *T*).

For each pair (algorithm *A*, time limit *T*) and for the set Total, we calculated the average ratio for each value of *H* and *R* over all 500 permutations. Figures 9 and 10 show the curves of the average ratio values obtained for, respectively, the parameters height and average reversal length.

Finally, to compare the distributions of height and reversal values obtained for the set Total with the distributions obtained by the algorithms, we performed a Kolmogorov-Smirnov test. As the sets of traces are very big (millions of traces), we generated for the set Total and for each pair (*A*, *T*) distributions of 5000 values (height or average reversal length) respecting the average ratios observed on the 500 permutations. These generated distributions were compared with the statistical test.

For the parameter height, the distributions produced for the pairs (DFALT, 36s), (DFALT, 30s) and (DFALT, 24s) were considered to be similar (or almost similar) to the distribution of the set Total with p-values, respectively, equal to 0.94, 0.46 and 0.04. In the case of the parameter average reversal length, the distributions produced for the pairs (DFALT, 36s), (DFALT, 30s), (DFALT, 24s) and (SWA6, 36s) were considered to be similar (or almost similar) to the distribution of the set Total with p-values, respectively, equal to 0.71, 0.25, 0.03 and 0.02.

The curves of the proposed algorithms exhibit different levels of approximation to the reference curve (Total) depending on the time limit imposed. The algorithm RA enumerates less traces and, consequently, is more distant from the reference curve. On the other hand, the algorithm DFALT enumerates more traces and has the best approximation to this curve. Considering the executions of the algorithm SWA, we have that SWA6 is the one that is closer to the reference curve.

Qualitatively, we can see that except for algorithm RA, the distribution curves tend to approximate the reference curve as we increase the time limit. This may give an indication that the algorithms DFALT and SWA can produce unbiased sets of traces with respect to the distribution of the height and average reversal length.

Naturally, as the time limit gets closer to the total time necessary to enumerate all traces, we expect that the algorithm DFALT gets closer to the distribution observed with the complete set. This is confirmed by the statistical test.

In the case of the non-deterministic algorithms, we cannot guarantee that the sampling will have the same property for small or big permutations. Nonetheless, Figures 9 and 10 show that the curve SWA6 gets gradually closer to the reference curve. Specifically in the case of the average reversal length, the Kolmogorov-Smirnov test confirms that the distribution of traces enumerated by the pair (SWA6, 36s) approximates the reference curve, with a p-value of 0.02.

## Conclusions

In this work, we proposed three different algorithms for the partial enumeration of traces: RA, DFALT, and SWA. Designed for processing big permutations, all proposed algorithms are able to do a partial enumeration of traces for permutations which cannot be processed by the actual algorithms for total trace enumeration, that is, BFA and DFA with or without permutation grouping.

The algorithms DFALT and SWA are based on the algorithm DFA. Thus, they inherited the inability of working with most of the biological constraints implemented by Braga *et al.*[21,22]. However, the algorithm RA can be easily adapted to consider these constraints.

Among the three proposed solutions, the algorithm SWA is capable of producing a number of traces higher than the ones produced by the other two algorithms when the same time limit is imposed during the processing of big permutations.

During our tests, we worked with time limits between 6 and 60 seconds. However, it is not an easy task to determine the time limit which should be used to produce a good sampling of the total space of traces which sort a given permutation.

One alternative could be to adopt other types of stopping criteria. For example, the algorithm could stop after achieving a fixed number of enumerated traces, or a fixed number of repeated traces (traces which were already enumerated). Nevertheless, these kind of criteria would be subjected to the same problems as the time limit criterion with respect to the guarantee of obtaining a uniform sampling.

A more advanced solution could involve a detailed analysis of the space of solutions of traces to determine a way of calculating the expected total number of traces. As a by-product, we could determine a percentage of the expected number of traces and use it as a stopping criterion.

In our tests, we could observe that the number of traces grows exponentially according to the ratio *d*/*n*. Nevertheless, to predict the number of traces which sort a permutation is an open question which requires more investigation. A deeper study about the characteristics of the permutations must be conducted with the aim of obtaining, if possible, a formula to calculate the expected number of traces of a permutation. In this direction, the results obtained by Braga and Stoye when analysing the solution space of sorting by DCJ operations [9] may provide some insights.

The difficulty of analysing the quality of the sampling for big permutations is that, for now, we are capable neither to calculate the expected total number of traces, nor to predict the general distribution of the complete set of traces just by looking at the permutation. The software MC4Inversion, written by Mikls and Darling [18], could be used to estimate a lower bound for the number of traces. To do this, we could get the estimated number of optimal solutions given by the software and divide it by *d*(*π*^0^*π*^*d*^)!. In this estimation, we assume that every trace contains only non-overlapping reversals. Obviously, this is not true and, in fact, the real number of traces can be much higher than this estimated lower bound.

We conducted tests with small permutations and we could see that the sets of traces partially enumerated by the algorithms DFALT and SWA have distributions that get closer to the distribution observed for the complete set of traces when we increase the time limit. This may give an indication that these algorithms can produce unbiased sets of traces, at least in relation to the distribution of height and average reversal length.

The height of a trace does not have a direct biological meaning but it provides some evidence of the complexity of the solutions that it represents. Traces with high height group solutions that have a high number of reversal overlaps. For example, some groups of bacteria evolve mainly through symmetrical or almost-symmetrical reversals relatively to the replication terminus. In this case, we could expect that the occurrence of small reversals contained inside big ones exhibits a ratio bigger than the one observed when the position of the reversals are not restricted. As a consequence, we would then also expect the solution traces to have low height values.

The average reversal length can be an important aspect in genome rearrangements. The algorithms RA and SWA show a tendency for losing traces that have high average reversal length. However, if we know that the target genome is subjected to reversals of small or intermediate sizes [26-29], the deficiency of these algorithms becomes a minor issue.

Independently of a biological meaning, the parameters height and average reversal length represent measures that are easy to compute and that can be used in the evaluation of the quality of a sampling.

## Competing interests

The authors declare that they have no competing interests.

## Author’s contributions

Implementation and test analyses were performed by CB. All authors participated in the discussions. The manuscript was written by CB with major contributions by MFS and ZD. All authors read and approved the final manuscript.
